# “I spend more time on the ecosystem than on the disease”: caring for the communicative loop with everyday ADM technology through maintenance and modification work

**DOI:** 10.1007/s00146-024-02109-5

**Published:** 2024-11-15

**Authors:** Sne Scott Hansen, Henriette Langstrup

**Affiliations:** 1 Department of Communication, University of Copenhagen, Karen Blixens Plads 8, 2300 Copenhagen S, Denmark; 2 Department of Public Health, University of Copenhagen, Øster Farimagsgade 5, 1014 Copenhagen K, Denmark

**Keywords:** Body, Embodied technology, Automated decision-making (ADM), Human-in-the-loop, Communication, Maintenance, Anticipation

## Abstract

Automated decision-making (ADM) systems can be worn in and on the body for various purposes, such as for tracking and managing chronic conditions. One case in point is do-it-yourself open-source artificial pancreas systems, through which users engage in what is referred to as “looping”; combining continuous glucose monitors and insulin pumps placed on the body with digital communication technologies to develop an ADM system for personal diabetes management. The idea behind these personalized systems is to delegate decision-making regarding insulin to an algorithm that can make autonomous decisions. Based on interviews and photo diaries with Danish “loopers”, this paper highlights two interrelated narratives of how users have to care for the loop by *maintaining* a stable communication circuit between body and ADM system, and by *modifying* the loop through analysis and reflection. It shows how the human takes turns with the ADM system through practical doings and anticipation to safeguard continuous management of chronic disease.

## Introduction

*Now that I have 300 measurements a day, I spend much less time on the disease than I used to. The system I’m running now, it’s, uhm, it’s a classic loop on an iPhone. […] I can look at my [Apple]Watch in case I need to do something about them; I don’t need to do anything about diabetes (Jesper).*

What the participant Jesper is referring to in the above quote is the phenomenon of “looping” that is practiced by some people living with diabetes. Looping refers to the use of technological devices set up as “closed loop systems” on one’s body to facilitate the tracking and management of diabetes, thereby helping users live a better life with the disease. Closed loop systems are automated decision-making (ADM) systems that involve a continuous blood glucose monitor (a “CGM” sensor placed on the body) communicating with an insulin pump (a computerized insulin delivery device placed on the body) that makes autonomous decisions and regulations regarding how much insulin to inject into the body and when. This happens via a personalized algorithm operated from a smartphone app that the user programs based on open-source code and connects to open-source hardware devices. Via the user’s smartphone or a connected smartwatch, device communication can then be monitored in data as the basis for how the algorithm operates and makes personalized decisions. Depending on the system used, sometimes a device (e.g., a “MiaoMiao”) is needed to bridge the communication between the sensor and the app, and a “RileyLink” to bridge the pump and the app. However, new versions of the CGM sensor are already being developed and brought into use. The “FreeStyle Libre 2”, for example, facilitates direct communication between the body and the app without the use of a MiaoMiao. Likewise, new versions of insulin pumps are being brought into use, such as the “Omnipod Dash” which allows direct communication between the insulin pump and the app without the need for a RileyLink. Both new versions have recently become available in Denmark where the current study was carried out. However, these types of products are mainly developed by private American companies, and their availability will therefore vary in different places and health systems. When users connect these devices, they allow them to communicate automatically with their body in an internal, closed loop. When used this way, the ADM system for looping constitutes what is referred to as an artificial pancreas, a technological substitute for the biological pancreas, which would normally produce the insulin needed to regulate the blood sugar levels in the body without any assistance. These open-source ADM systems are, therefore, typically referred to as do-it-yourself (DIY) artificial pancreas systems (APS), as they are the result of patient innovation and the users setting up their own closed-loop system outside the purview of healthcare authorities and professionals. Ideally, by setting up a closed-loop system, the human user can delegate most of the complicated decision-making regarding insulin to the autonomous algorithmic and sensor-based system, which “simply works in the background like any other organ” (Garfinkel 2020: n.p.). However, the use of ADM for looping still requires constant and mutual attentiveness between the person, the body, and the technological devices (Wiedemann 2021). It relies on mutual autonomous and anticipatory practices that involve both the human and the system continuously adapting to each other.

The idea of allowing an algorithm to make vital decisions on behalf of a human, as is the case in looping, contrasts with the way larger institutions and governments have oftentimes advocated against delegating human decision-making to ADM systems (Scott Hansen 2022). For instance, the Ethics Guidelines for Trustworthy AI mention that “[h]uman oversight helps ensure that an AI system does not undermine human autonomy or causes other adverse effects” (European Commission 2019: 27). This “[…] may be achieved through governance mechanisms such as a human-in-the-loop (HITL) […] approach. HITL refers to the capability for human intervention in every decision cycle of the system, which in many cases is neither possible nor desirable” (Ibid.). However, what is still lacking from this picture is a detailed understanding of how the idea of involving “the human in the loop” is already being translated by individuals into concrete ways of living with ADM systems today. Rather than looking at AI or ADM technologies in themselves, we need to begin to look at the many contextually bounded “*ways* in which a certain technology—which may be far less sophisticated or ‘intelligent’ than deep learning algorithms—is *inserted* within a decision-making process” (Chiusi 2020, p. 4, emphases added). This paper responds to recent calls to “re-humanize algorithmic systems” (Ruckenstein 2023: 1241) by exploring “how humans are involved and thereby implicated in such [technological, ed.] processes” (Ibid). It does so by taking an empirical starting point in the case of looping in diabetes. We ask *how humans who engage in looping are present in the loop as they embody their ADM system in everyday life, what it takes to be there, and how it is experienced*.

By way of narrative analysis, we highlight how looping works as a complex communication circuit between the human and ADM, which needs to be cared for through maintenance and modification work. These narratives are manifested in everyday experiences of being in and taking care of the loop by maintaining a stable communication circuit between body and technology. At the same time, they also recount everyday experiences of caring for the loop through modifications of personal data and preferences in relation to their own physical body and unique circumstances. As such, we extend what Wiedemann (2021) has described as a move from caring for the body to caring for technology by showing how the latter is manifested in two different but interrelated ways when embodying and engaging with ADM; namely, how users have to care for the communicative loop with everyday ADM through maintenance and modification work. Thus, the narratives in this paper provide insights into the embodied aspects of experiencing everyday human–ADM relations as they play out in practice.

## Embodying everyday ADM systems

Up until now, discussions of the role of ADM in society have tended to focus on what automation does or might do to people instead of what people are already doing with automation (Pink et al. 2022a: 4). This former perspective has been instrumental in setting an agenda for how to think about the human role in relation to ADM. Discussions often lean towards perceptions of the ethical risk or practical benefit of delegating a part of or the whole decision-making to the technology. Such discussions often agree that we need to include the human in the loop in a certain way, retaining our role as active decision-makers. At the same time, they have kept alive a fear that ADM might possibly end up overruling human decision-making (e.g., Scott Hansen 2022; Lupton 2021; Bareis & Katzenbach 2021). If we want to take human agency seriously, we need to shift the discussion towards what people are already doing with ADM, thereby moving it from focusing on the effects of ADM to seeing humans as its co-creators.

If we turn our perspective towards how ADM is already used by ordinary people in their everyday lives, a growing body of literature is starting to unpack the multiple ways in which humans are active participants and communicators when embodying and engaging with ADM, sometimes referred to as "everyday automation” or everyday ADM (Pink et al. 2022a, b). For instance, in her study on looping, Wiedemann (2021: 32) points out that despite various technologies taking over more of the care workload from humans, “digital improvements do not, in fact, lead to the pairing of passive subjects with active technologies that take over the reins of responsibility” (Ibid). Instead, humans are oftentimes highly involved, not necessarily as active decision-makers, but rather as active maintenance workers of the specific devices or technologies they use. Wiedemann (2021) further identifies how loopers “constantly need to be in a state of readiness in order to repair, replace, recharge or reconnect the devices that help attend to the disease” (Ibid: 33). She argues that this has resulted in patients’ care for the body now being replaced by a care for the technology they are using to manage their disease, as the algorithms “must be navigated” and kept in “delicate balance” by patients (Wiedemann 2021: 51,52). This indicates that humans are working towards maintaining a communication circuit between their bodies and devices to facilitate ADM. Looping depends on human involvement and management of internal communication between the person using the system and the system itself because a mix of medical devices, consumer devices, and advanced algorithms interact and make decisions autonomously with unreliable bodies. Relatedly, Forlano (2023a: 25) has developed the notion of “cyborg disability” that acknowledges not only human, but also technological disability, unreliability, and imperfection. With this notion, she points to how “[t]he fragile arrangement of human, machine, biotechnological, and analog things make life possible, and also sometimes, quite impossible. While technologies offer new affordances and modes of living, they also introduce complexities that require attention, care, and maintenance” (Forlano 2023a: 26). As she continues, based on personal experiences of living with diabetes technology, “[h]abits, routines, practices, and even notions of selfhood and subjectivity are reshaped around seemingly small modifications. Often, I am not sure whether I am taking care of these devices or they are taking care of me” (Ibid.). Kaziunas et al. (2017) also find that people who use technologies for looping carry out the “continuous work of holding diverse subjects, objects, bodies, processes, and institutions together” (Ibid.: 15). They thus underline how “DIY health care is neither something done “by yourself” nor easily reduced to an “act of innovation”” (Ibid.). Rather, they point to how it is the partnership between humans and devices that ensures successful looping. As the authors show, one central aspect of understanding these partnerships concerns “[…] the materiality of devices but also […] data’s relationship to flesh and blood, skin and muscles” (Ibid.: 20). Schwennesen (2019) similarly explores the embodied aspects of engaging with ADM technologies for physical rehabilitation and how people are involved in practical tasks with algorithms. She argues that algorithms’ “agentic capabilities” are the result of the human’s involvement in building, maintaining, and repairing “enabling connections between bodies in-motions and professionals in arrangements of care” (Schwennesen 2019: 176). She points to how the system’s suggestions are based on its interaction with bodily movements picked up by body sensors and translated into data.

Taken together, these studies highlight the daily and manual work that goes into engaging with everyday ADM systems. Although the literature acknowledges humans as active participants and sense-makers rather than passive subjects, they are mainly described as the ones who take care of rebuilding, restoring, and repairing devices. However, this literature risks overlooking the more reflexive aspects that play a crucial role in people’s experiences of embodying and engaging with everyday ADM. These aspects involve how humans oversee, facilitate, analyze, modify, and reflect on the communication between the body and devices. If we do not take these aspects into account, we risk reducing the human to someone who is limited to the practical, physical, and manual work with ADM. At the same time, we risk overlooking the personal stories, circumstances, and emotional reasons why people choose to engage with ADM in the first place. What we need to understand are the multiple ways people are present in the loop, as they embody everyday ADM and begin to engage in different forms of communication with their devices.

## Communicating with everyday ADM systems

### Turn-taking in looping

The concept of turn-taking offers a theoretical tool to understand the continuous communication circuit between the user and ADM system in looping. Originating from linguistics and conversational analysis (Sacks et al. 1974), turn-taking has typically been used to analyze interactional patterns of how humans alternate between communicating and listening to each other, sometimes using various communication technologies. Herring (1999), for instance, has described how the use of computational devices allows for a “more efficient form of turn-taking” because communication from the various participating actors can happen simultaneously by data being automatically tracked and stored (Ibid.: 8). Data then work as a textual “record of the preceding interaction” (Ibid.). Herring (1999) also points to how interactions with and through these systems are marked by moments of breakage resulting in the communication not developing linearly. This requires the user to work more actively towards maintaining communication, such as by using “follow-up turns” to maintain sequential coherence (Herring 1999: 5). As Sacks et al. (1974: 723–724) argue, repair and maintenance mechanisms “exist for dealing with turn-taking errors and violations”, and “[…] are intrinsic to the very system whose troubles they repair”. This is particularly notable in how humans are involved in the loop of DIY APS. In this case, turn-taking presents a useful metaphor to specify how humans are *communicatively* involved in the loop with everyday ADM, and what a human can do in his or her turn that the machine cannot do and vice versa. While some studies within the human–robot interaction (HRI) and human–machine communication (HMC) fields have investigated turn-taking patterns in humans’ direct interaction with AI and ADM technology, such as virtual agents, (social) robots and chatbots that are specifically designed to communicate with people (e.g., Skantze et al. 2014), these forms of interactions are typically very different from the ways humans interact with embodied everyday ADM systems. Embodied everyday ADM is usually not intentionally anthropomorphized but resides in medical or consumer devices, such as smartphones or smartwatches, where they are unassumingly woven into people’s daily routines and rarely come to the fore.

When people embody ADM in looping, they create a communication circuit that works in the background of the body by interacting synchronously with it continuously throughout the day. The communication circuit works as a “[…] kind of call-and-response system, where an insulin pump communicates with a CGM in real time to automatically adjust insulin according to fluctuations in blood-sugar levels” (Garfinkel 2020: n.p.). The insulin pump is a computerized device that can inject insulin into the body and is usually worn on the stomach. The CMG, i.e., the continuous blood glucose monitor, is usually worn on the upper arm and works as a sensor with a small needle that generates data about the blood-sugar levels in the body. For the two devices to communicate, the user has to build an algorithm that can be accessed on the smartphone. The algorithm then makes autonomous decisions based on data from the CGM and communicates these decisions to the insulin pump. This all happens via two devices; the MiaoMiao (placed on top of the CGM sensor) and the RileyLink (a portable device that needs to be kept close to the user and its phone when moving around). The MiaoMiao communicates with the sensor and the algorithm, and the RileyLink with the insulin pump and the algorithm.^1^ These devices must be kept at a certain distance between the body-worn sensors and the smartphone for the loop to function. Together, they constitute an internal body-system communication circuit, where the body and the ADM system take turns communicating and responding to one another. Communication in the use of everyday ADM for looping is thus based on turn-taking, both between the body and the two body-worn devices, and in-between these two devices and those that are linking them to the algorithm on the smartphone.

### Anticipating turns in the loop

Turn-taking implies that communication, in the form of statements or actions, anticipates what may happen in the next communicative turn. As such, anticipation is central to looping where the user might need to step in, anticipate what will happen next, and thereby influence the communicative outcome in the loop. The anticipation of turns in the loop, whereby the user involves herself by altering and modifying the communication that takes place between body and ADM system, relates to Adams et al. (2009: 246) definition of anticipation as the state of “thinking and living toward the future”. They argue that “[…] anticipation is not just a reaction, but a way of actively orienting oneself temporally” (Adams et al. 2009: 247). It is a way of actively embodying time, both the now and the future, thereby pointing to the sensed, lived, and felt aspects of anticipation. As Adams et al. (2009: 249) describe it, “[a]nticipation is not just betting on the future; it is a moral economy in which the future sets the conditions of possibility for action in the present, in which the future is inhabited in the present”. In that way, it becomes, as Adams et al. (2009: 250) describe it, “a strategy for avoidance of surprise, uncertainty and unpreparedness, but it is also a strategy that must continually keep uncertainty on the table”. Anticipation has also often been associated with technologies such as ADM or AI that are sometimes referred to as “anticipatory devices” (Pink et al. 2022b). However, Rahm & Kaun (2022) note that historically, the portraying of anticipatory forms of interaction and communication between humans, ADM, and related technologies, lie very far from the contingent ways in which humans actually anticipate these technologies when interacting with them in practice. As they note, such interactions are oftentimes “[…] characterized by failures and dead ends” (Rahm & Kaun, 2022: 23). Such everyday uncertainties are part and parcel of living and dealing with the “digital mess” resulting from our interaction with a range of digital systems, including ADM systems (Pink et al. 2018). In this context, “data anxieties” and trust are examples of “anticipatory modes” surrounding people’s experiences of living and interacting with their data (Pink et al. 2018: 1). They manifest themselves as everyday improvisatory actions, such as duplication, archiving, and saving routines of data from our devices (Ibid.). This furthermore occasions the development of personal know-how as part of one’s routines of engaging with “everyday digital data” and digital objects (Pink et al. 2018: 10). They argue that everyday digital data are “ongoingly produced and made” because they are incrementally and “continually modified by their users” and because data are automatically and continuously added “with varying degrees of human intervention” (Ibid.). In addition, they point to how anticipatory modes are rooted in embodied sensory experiences and not necessarily in cognitive decisions. For instance, trust in one’s data refers to a sensory anticipatory mode “[…] of living in a world of uncertainties that ‘feels right’, a sense of control in a space of uncertainty” (Pink et al. 2018: 3), “a sensation often achieved through the accomplishment of mundane everyday routines” (Pink, 2021, p. 196). Anticipation is based on embodied knowing and feeling and entails unspoken communication between humans and ADM that, in looping, become a matter of life-or-death.

## Methods

As part of a bigger study on body and tracking technology, we recruited three loopers in their 20–40s in May of 2022. The first author conducted three iterations of semi-structured photo diaries combined with in-depth semi-structured elicitation interviews from May to September 2022,^2^ resulting in nine interviews and photo diaries that form the basis for the following empirical analysis. The first iteration of photo diaries and interviews was dedicated to getting to know the participants and gaining an insight into how looping was implemented into their daily lives. The second iteration focused on the collaborative and embodied aspects of their experiences of the relation between the human and the system. Lastly, the third iteration circled around questions of bodily communication and data. In the first photo diary, participants were asked to take pictures illustrating something about themselves and their daily lives, e.g., what their day had looked like, which topics they were particularly concerned about at the time, or some apps they had been using. In the second round, participants were asked to take pictures of situations where they had been using their looping system. Finally, in the third round, participants were asked to take pictures of situations where they had experienced their system and/or their data as particularly meaningful to them.

The tasks for the photo diaries were open-ended with flexibility for the participants to interpret and respond to the diary task in their way. The photo diaries provided us with insights into how people with diabetes engage with and make sense of the ADM systems for looping. In particular, the embodied, sensed, physical, and material aspects of living with ADM as an extension of the body became evident in the photo diaries, thereby opening for further dialogue in the interviews that took into account these dimensions of how the human user was involved in the loop. In addition, the photo diaries afforded a glimpse into the daily lives of people living with diabetes and ADM. The diaries also revealed some of the daily practicalities and trivialities that could otherwise have gone unnoticed in the interviews yet were of major importance for the experience of living closely entangled with these systems in and on the body.

Based on this material, the following analysis presents two narratives illustrating variations in how people engage with looping as a communicative system. As such, the analysis focuses more on the “told” rather than the “telling” itself (Riessman 2005: 2). Bigger chunks of the transcribed interview material are analyzed along with the photo diaries, allowing us to gain insight into experiences expressed as personal narratives; “storied ways of knowing and communicating” in the form of “extended accounts of lives in context that develop over the course of single or multiple interviews” (Riessman 2005: 1). As narratives are “[e]mbedded in the lives of the ordinary, the marginalized, and the muted” (Langellier 2001: 700), this is where we need to look for experiences of everyday ADM.

## Narratives of being in and caring for the loop with DIY APS

Looping is often referred to as “automation” of diabetes self-management, and the future of diabetes management is expected to provide a fully automated artificial pancreas system (Lewis 2021). At the same time, looping currently depends on the user’s practical setup, maintenance, and modification of the system in relation to their own physical and biological body. In this analysis, we focused on the everyday experiences of embodying and engaging with looping as a communicative system: What do users have to do to enable and maintain the circuit or—in emic terms—the loop of communication between the body and the various devices involved? How do users experience this engagement, and how do they make sense of their relation with a technical system? While it is well known that the initial setup of the system involves the user—hence the popular reference to DIY—we have focused on how users engage with the system once set up and “running”.

The overall findings from the interviews and photo diaries concerned the ways in which users have to care for the loop, as the “automated” loop is deeply dependent on human involvement. We found that there are two main narratives of how users are involved in and care for the loop: (1) by maintaining a stable communication circuit between body and system, and (2) by modifying the loop through analysis and reflection. We see different emotional experiences and ways of making sense among the participants associated with each of these modes of caring for the loop. These are related to certain turn-taking mechanisms and anticipatory practices with ADM, shaping the way the users were involved in the loop.

### Caring for the loop through maintenance: keeping a stable communication circuit

**Fig. 1:**
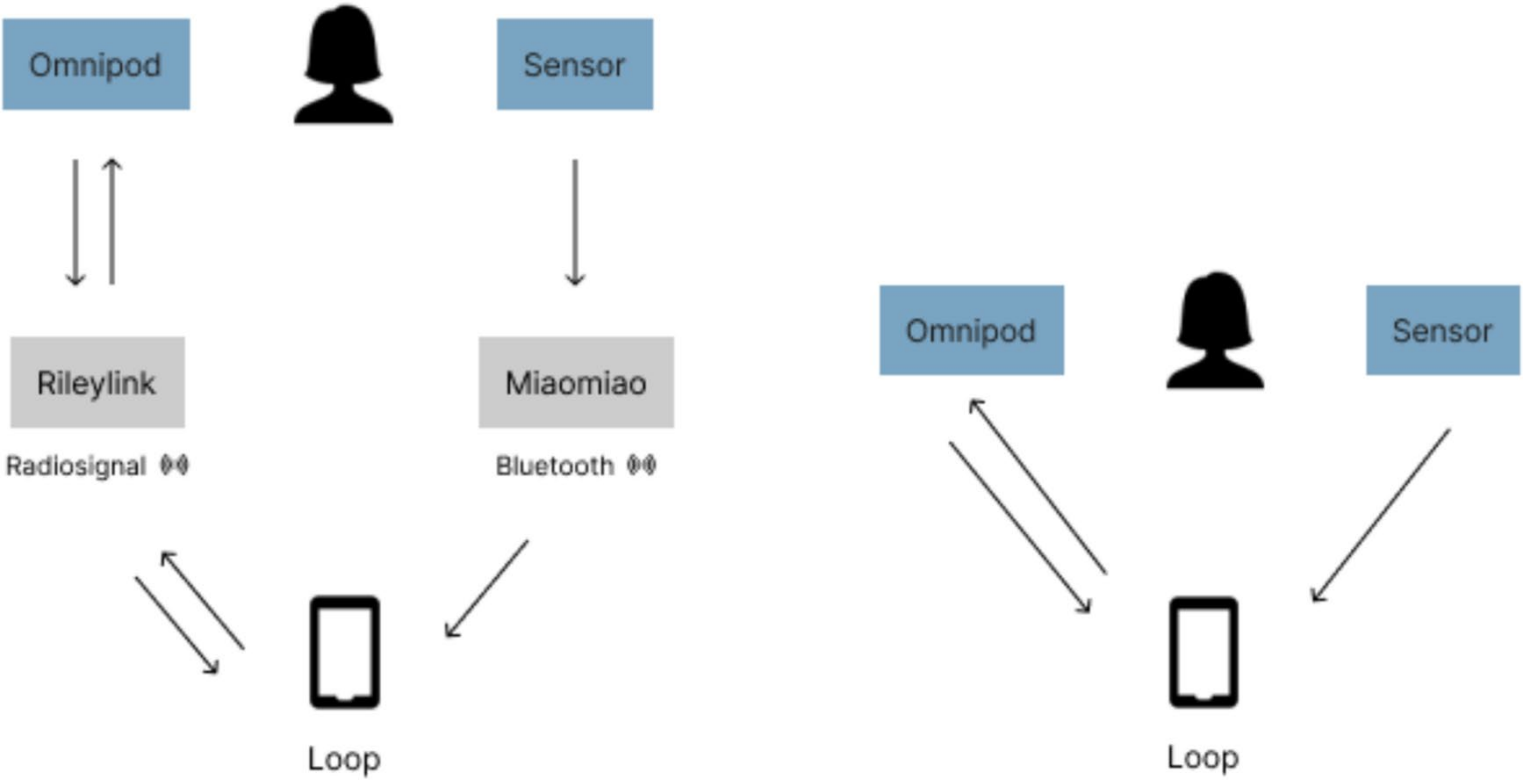
Illustrations made by the participant Hannah, on the left, showing how her closed-loop system used to work, and, on the right, how it now works based on newly developed devices

**Fig. 2:**
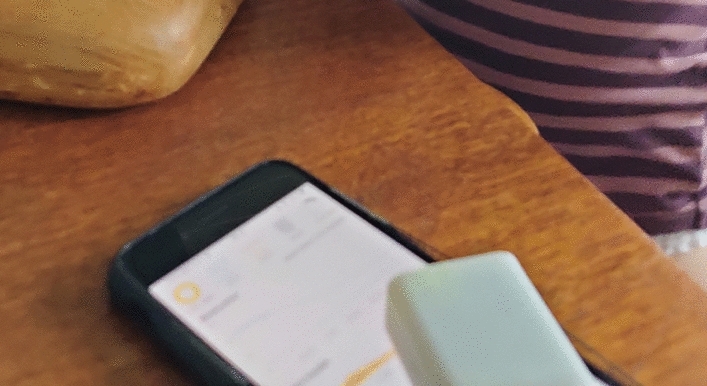
RileyLink hovering over the ADM app on smartphone

**Fig. 3:**
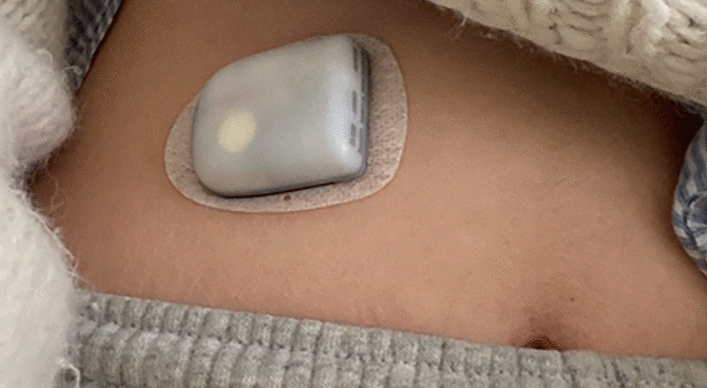
Insulin pump (Type: Omnipod)

Participants described their relationship with the loop as characterized by almost synchronous turn-taking patterns of body-device and device-device communication. The turn-taking patterns begin with the interstitial glucose markers under the skin that are picked up by the body-worn glucose sensor (CGM). Sometimes with a small delay, it is encoded as data and picked up by the MiaoMiao communication device placed on top of the CGM. Next, the data are transferred via Bluetooth to the autonomous algorithm installed in the form of a personally programmed app on one’s private phone. Based on these data communicated from the body, the algorithmic system, in turn, automatically communicates back to the body via the RileyLink and insulin pump, resulting in new interstitial glucose markers once again being communicated by the body and picked up by the sensor. Looping thus always involves several devices worn directly on the body, close to it, and on top of each other all at the same time, but the exact types of devices might vary slightly across the participants and over time as new versions are developed and made accessible. For example, Fig. 1 below, made by participant Hannah, demonstrate her transition from using five devices to using a simpler loop consisting of three devices that she had just set up at the time of our last interview. This change of setup was made possible because new versions of devices had become available in Denmark. In her current setup, she is using a new version of both the CGM and the insulin pump (specifically a FreeStyle Libre 2 and an Omnipod Dash) that communicate directly with the Loop app on her phone. The reduction of devices constituting the loop means fewer devices will have to connect with other devices, and be maintained, which was one of the main reasons for Hannah’s wish to change the setup. However, in any of these loops, users have to put work into ensuring an efficient, continuous, and stable communication flow between the body and each of these devices to make sure that the loop is constantly running for the algorithm to make decisions on behalf of the human. In any case, the goal of looping is to keep the conversation—the exchange of turns—in the communication between the body and devices going. Several of the pictures shared by the participants also showed the arrangement of different devices in close proximity to each other and to the participant’s body to ensure proper connection, efficient turn-taking, and thus continuous communication between all the technological entities and the body (Fig. 2 and 3). This was described by the participants as intrinsic to looping because it aims at creating a technological pancreas that can work autonomously in the background by communicating in an internal, symbiotic loop with the human body.

Emplacing devices on or near the body to ensure that they do not fall off, are uncomfortable, or lose connection is one care practice. Participants described their experimental approach to trying out places for the CGM and pump but also how the added elements for looping—the MiaoMiao, the RileyLink, and the phone—are emplaced and cared for: “I have bulky pockets, because I always have a RileyLink and a phone in there” (Jesper). Having the phone and the RileyLink in the pocket also has to do with ensuring “connection”. This is essential, as the MiaoMiao communicates via Bluetooth to the phone, while the RileyLink communicates via radio waves to the pump. Keeping the connection and communicative turns going between the body and the technological devices is part of Hannah’s morning routine: “Then I head for the shower and bring my phone and my little radio transmitter [RileyLink, ed.], so it won’t lose connection while I am in the shower”. The app running the algorithm will notify the user if it loses connection, in which case troubleshooting is needed. In a later interview, Hannah describes how she received numerous notifications in the app at one point, alarming her that it was not able to receive sensor data from the CGM. It turned out that the MiaoMiao had “crashed” due to saltwater seeping into it during swimming: “[…] then I opened it up and it was all rusty inside” (Hannah). The breakdown caused Hannah to pause her looping to try to get a different CGM from her health provider, which could communicate directly with her algorithm via Bluetooth, thus making the MiaoMiao redundant. Here, she explained how she had to apply for the CGM via her treatment center as not everyone can be granted the same types of devices, even if they exist on the Danish market:

I started a slightly more extensive process with my treatment center where I tried to get permission to get an upgraded sensor so that I don’t need the MiaoMiao. […] Libre 2, it’s called. The one I have now that you need to use with the MiaoMiao is an older generation, so it’s called Libre 1. (Hannah)

**Fig. 4:**
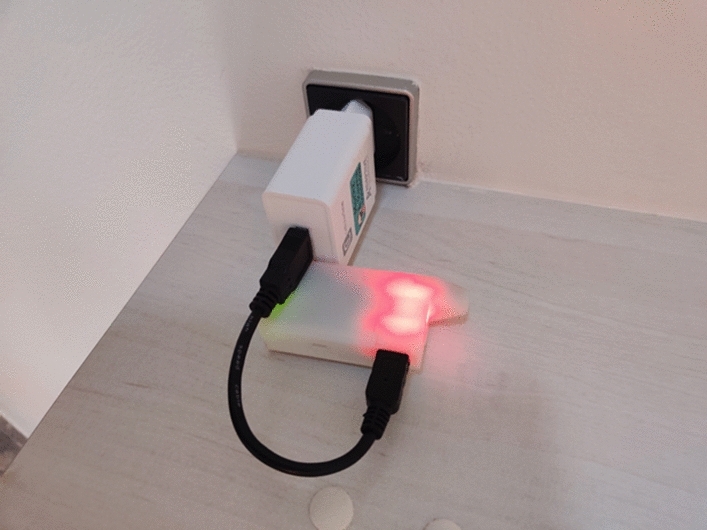
RileyLink being charged

The care for the loop in this instance also involved accepting time off from the loop to procure more durable and interoperable technology. At the same time, the quote shows how Hannah was taking a proactive approach to maintaining and making the communicative loop more efficient by upgrading to devices that could optimize the turn-taking mechanisms of the overall system. With a new generation CGM in place, Hannah is now able to ensure more efficient turn-taking between her body and the ADM system. The participant Sofia also described how alternative and rather drastic solutions are sometimes required if the connection is not working. She described how she once followed a tip shared in an online group that recommended placing her insulin pump in the microwave oven to stabilize its connection with the RileyLink, which ended up working again after the procedure. Her story illustrates the sometimes unconventional and DIY approaches users take to maintain the communicative loop. Tinkering, testing, and experimenting are necessary practices through which users express their care through maintenance work. However, another more common care practice concerns the regular charging of the MiaoMiao, the RileyLink, and the phone that users have to manage on a daily basis as these are all vital bridges between the body and the system (Fig. 4). As participant Sofia described it, simply making sure that charging routines are smoothly embedded into everyday life is part and parcel of making the communication circuit between the body and the ADM system work:

So, I always walk around with it and it has to, I mean, everything has to be charged. I think that's one of the things that’s a bit annoying, isn’t it? And especially with this MiaoMiao, because I can’t just take it off, so I’ll have to sit with a charger on it and then with a, what's it called... a power bank, because otherwise—that is, the charger cable that goes with it. It's this long, so you can’t sit next to a socket. (Sofia)

What Sofia describes is the work of charging devices attached to her body when using her embodied everyday ADM systems. Her expression of annoyance with what in some ways seem to be small and trivial practices, however, underscores an additional level of complexity in maintaining the system. Namely, Sofia needs not only to charge devices of the system but also make sure that the very maintenance devices themselves—such as portable power banks—are fully charged so she can, literally, charge herself on the go and maintain the functionality of the loop. This aspect—the importance of carrying power banks and having backup pumps and CGMs in case of system malfunction—was also described by the other participants. Jesper explicitly describes this paradox of how a system that makes living with diabetes a lot easier, at the same time adds levels of complexity to the experience of living with and managing diabetes: “[…] Loop always adds a layer on top where the consumer electronics actually becomes as important as the medicine itself, and thereby the risk and complexity increase, and the amount of backup equipment you have to carry with you grows accordingly” (Jesper). These examples highlight the physical and material aspects of the types of turns the system is taking in the loop when interacting with the user’s body, i.e., transferring, picking up, and broadcasting different types of bodily and technological signals, thereby converting them into data visualizations available for the user. They also show how turn-taking becomes a technical skill when it comes to humans’ interactions with everyday ADM systems, such as DIY APS that depend on the user developing the abilities to set up, fix, and maintain an advanced technological system independently. At the same time, turn-taking with DIY APS becomes a bodily skill in that it relies on the user ensuring a bodily connection to devices. This involves distinct bodily movements and proximities to devices in the surroundings as well as attachments and replacements of devices on specific body parts and areas to encourage a dialogue between devices and the body. Each part of the ADM system can expect its signals to be picked up and responded to within the system only when facilitated by equal turns taken in the communication circuit.

These descriptions of caring for the loop as a communication circuit thus reveal a constant awareness of bodily communication with the technical system. The descriptions also show that the users know what is needed for the loop to run autonomously and continuously. These care practices and the constant awareness—both supported by visualization of connections on the phone or smart watch and alarms from the system—are described as demanding: “I have to use energy to keep consumer electronics and medical devices running. You always have to have everything charged; you always have to have backups. […] There are many mental checklists every time I leave the house” (Jesper). However, the participants describe these practicalities and “mental checklists” as acceptable trade-offs, as they enable the ADM system to take over the “micromanagement” of insulin dosing, resulting in improved outcomes. Moreover, some of these practices are “normalized”, as they do not expose the disease of the individual to the surroundings:

I spend more time on the ecosystem than on the disease, and that’s, sort of, an advantage because the ecosystem is… it’s not… well, everyone is always looking for power for their phones, right? I mean, that’s a much more natural thing. No one will raise an eyebrow when you fiddle with a smart watch or sit and look at your phone. Well, at most they may think it’s a little rude, but that’s something completely different from sitting with medical equipment. […] So, in that way it’s, sort of, much more mundane. It’s about making the disease fade into the background. (Jesper)

In the quote, Jesper also describes how he himself experiences the practices as being less about disease management simply because other people do not see his practices as disease-focused. Hannah similarly describes how people have thought that she was doing online stock trading while on her phone in public rather than dealing with a health issue. Being overseer of an autonomous communication circuit via consumer technology normalizes disease management activities while at the same time disguising the effort and care required.

### Caring for the loop through modifications: managing personal data and preferences

As participant in and overseer of the communication circuit of the loop, the user also engages in continuous adaption and modification of the loop depending on analysis of data from the system or changes external to the circuit. Moreover, users base their modifications on their experiences, plans, and hopes for the near and far future. In these instances, the human–ADM communication is characterized by asynchronous turn-taking patterns between the human and the autonomous communication circuit consisting of the body and the above-mentioned devices. Throughout the day, users have to input data about what they (plan to) eat for the algorithm to adapt its programmed course of action to carb intake. As such, users alter the communication taking place in the circuit of body and devices by interacting with the ADM system through evaluations and modifications of the data and settings that the communication between the algorithm, the insulin pump, the CGM, and the body is based on. To do this, the human has to monitor and evaluate the system to know which additional data to add in the app for the algorithm to make more precise decisions. At the same time, users need to stay aware of bodily states, feelings, activities, and outside circumstances that play a crucial role in how the system regulates their blood glucose (BG). In this way, users have to work on anticipating the next communicative turn in the autonomous circuit by actively stepping into the loop and influencing future communicative outcomes in the ADM system through interaction with personal data and algorithmic settings. The goal is to help the algorithm make better future decisions, which means that users have to take turns in dealing with the uncertainty of the system by communicating certain information to it in advance. The participant Sofia describes how she takes turns in involving herself in the loop from the outside by interfering with the data when the algorithm, here referred to as the name of the app, “Loop”, requires guidance:

Loop calculates how much medicine I need. I just have to tell it how much I eat. Then it calculates the rest. If it for some reason doesn’t function, then I have to calculate [..] I actually find it difficult […] it’s because I am not a computer, but Loop is. (Sofia)

Sofia here describes the division of labor between herself and the ADM system as reasonable; she is needed for data input but not for doing the calculations, a task a computer does better than her. As such, she is actively involving and orienting herself temporally—either to the automatic or manual calculations—in an anticipation and modification process of managing diabetes. Here, present actions and circumstances of everyday life are carefully and continuously considered in relation to future bodily communication and health outcomes related to the use of the system. This shows how Sofia is asynchronously involved by reflecting on and inputting data into the synchronous turn-taking that is already taking place between her body and the devices. Anticipating turns and modifying the loop by monitoring, analyzing, reflecting on, and manually editing or adding data in the system are other care practices characterizing the way in which the human user is taking turns when communicating with the embodied ADM system. However, the ADM system does not always work perfectly, despite the input and the care involved in making the circuit run. The participants all describe how this may cause frustration, particularly if expectations of the system were high to begin with. For example, Sofia explained in the first interview how she had at one point stopped using the ADM system, because it did not live up to her expectations and made her stressed:

I couldn’t keep myself from looking at my numbers and on top of that I didn’t feel it lived up to my expectations. It might have been because other people in the Loop-group [an online community of loopers, ed.] were really good at describing their successes with using the system and I got jealous. I could see how much they had improved, and I hadn’t. (Sofia)

After some time, Sofia realized that it might be worth trying again with different settings:

I started experiencing higher BG at night, and I didn’t know how to get them down again. Then I thought “okay, maybe I should try again with some new settings and see if that works” […] It has worked really well this time […] I made myself some better settings. (Sofia)

These examples illustrate how Sofia analyzes and reflects on her results and her relation with the system. These reflections also involve emotional aspects such as the stress and disappointment Sofia experiences. Such emotional aspects can be considered “anticipatory modes” (Pink et al. 2018), rooted not only in cognitive decisions and reflections of her engagement with ADM but also in embodied sensory experiences. Sofia encounters and engages with looping as an ADM system through the daily unspoken habit of constantly picking up her phone to look at the data communicated to her in the Loop app and through experiences of how she is feeling physically in her own body at night. Most importantly, the experiences, reflections, and analyses become occasions for making changes to the system to improve future results and experiences of engaging with the system. For example, in reflecting during our second interview on the importance of continuously considering and modifying her settings, she further describes the aspect of trust:

[…] that’s also what’s crazy; it’s really just an algorithm. These are some settings that I myself… have set up, but they give me a sense of security. So, knowing that you don’t have to get up at night to see how your diabetes is doing. I know people who do that. They set alarms because they are nervous about whether their blood sugar is too low… and this also makes me think of when I was younger and had been to a party where I experienced an insulin shock at night, and there I think… so, I don’t trust 100% that it wouldn’t happen again, but there is less risk… (Sofia)

As she expresses in a later interview,

there’s an algorithm inside the loop that knows—because I’ve also entered my different settings for insulin sensitivity and all that stuff—it knows ‘Sofia, she needs just two extra units of insulin’, and it actually does that by itself—but only because I have the RileyLink that makes the pump, the phone, and the blood glucose sensor talk together. (Sofia)

These considerations resonate with Pink et al.’s (2018: 3) description of “living in a world of uncertainties that ‘feels right’”, and a way of having “a sense of control in a space of uncertainty”: More specifically, trust here is associated with the feeling that the algorithm “knows” her. At the same time, however, the quote shows Sofia’s role as a modifier of the system working as the very basis for the system to get to know her in the first place. Moreover, it further emphasizes her practical role as a maintainer of the many devices constituting the communication circuit. It thereby also illustrates how modification is closely intertwined with the maintenance of the loop. In relation to the above examples, several of the participants also reflected on how the system should not be in control and that they were able to override it or ignore it if they chose to:

It is just a technical system, right, so sometimes I diverge from what it suggests. It is very much a situated thing, how much I take control over the app and how much I let it [my treatment, ed.] depend on the calculations it provides. (Hannah)

**Fig. 5:**
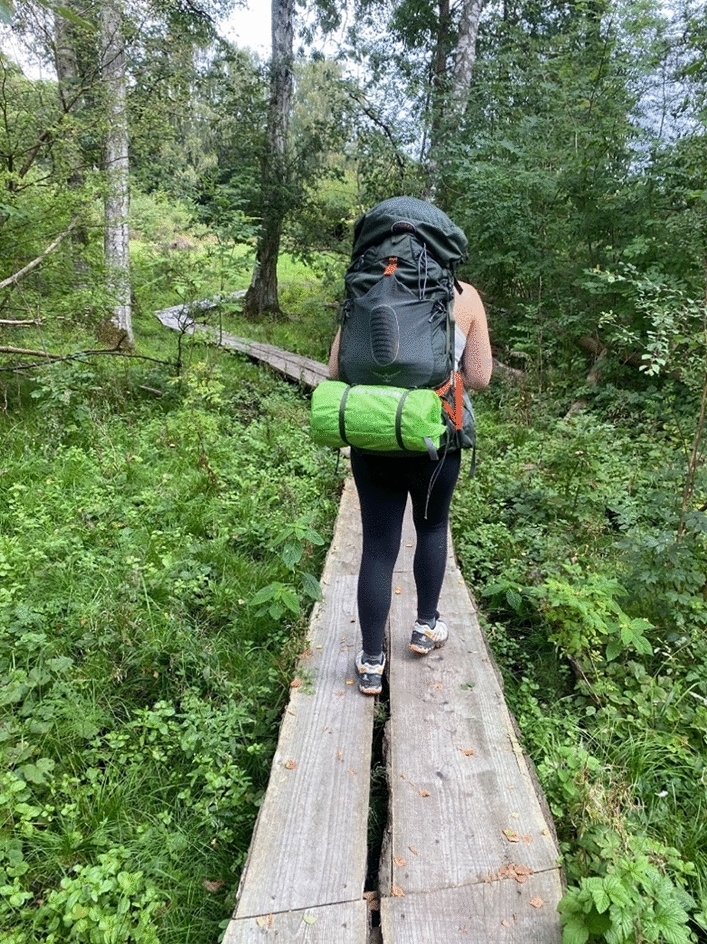
hiking with new settings

Hannah also points out that her body is not a stable entity, and changes are therefore necessary when using an automated system: “Despite trying to set it [the treatment, ed.] on an automated formula, you are just a body that develops and changes all the time” (Hannah). Taking care of the loop thus also depends on distinguishing between what Loop is and can do, and what the human user needs to do, resulting in some turn-takers in the loop with ADM being considered more equal than others: “What Loop cannot do is regulate the settings” (Jesper). Sofia, for instance, describes how she went on a hike with changed loop settings to accommodate for the more strenuous physical activity and lack of access to food and immediate medical assistance. She set what she referred to as “hiking settings”, where a higher BG is preferable to too low BG, particularly during the night (Fig. 5). She describes, in the third interview, how her continuous and cautious modifications of the system settings on previous hikes eventually led her to completely turn off the system during hikes, although she did explain how checking in with data and modifying settings initially helped her “see a pattern” in her sensor data. As she said,

I’ve tested some different settings but […] instead, I just switched off my pump completely for, perhaps, the first two hours of each trip because I’ve been able to see that this is where I get low blood sugar. […] it’s given me some more peace of mind knowing that I’ve now turned it off completely. It doesn’t run at 50% or anything, but now I can relax completely because what if the 50% setting inside the loop was not enough? (Sofia)

To Sofia, it “felt” better to intervene and modify the functioning of the loop by turning off the system completely because,

[…] then I know exactly what’s going on and it’s probably also because I haven’t familiarized myself enough with all these settings, and that would also require me to—on each trip—almost do a kind of investigation: What happens to my body when I put it [the settings, ed.] down by 40%? 50%? 60%? And I think that would be a bit too difficult. (Sofia)

Thus, the user’s involvement and care for the loop are manifested in everyday improvisatory actions and modifications of the communication circuit where the user communicates with the system via settings or data inputs about outside factors that are likely to affect the systems’ decision-making processes. As such, learning how to change settings may be a matter of trial-and-error and may also involve engagement with online information and fellow loopers in the online looping community. Through such processes of active, explorative, and creative engagement with communities and online information, knowledge becomes embedded into users’ offline tinkering with their devices:

Then I looked at different forums. There’s both a website where you can look in their FAQ for guides, but sometimes I also had to scroll a lot on an international Facebook page before I found an answer. And often then... well, I've spent a lot of time searching for the problems I was having and then scrolling again, and there just wasn't anyone who had experienced the same thing as me—at least not anyone who had written about it. So sometimes, I've had to... well, put together small parts from different posts to figure out what to change in my case. (Sofia)

Even if the ADM system is experienced as helpful, efficient, and even “essential”, participants described themselves as being the ones in control. And when Sofia was asked if she saw her relation to the ADM system as one of collaboration, she replied that “dominatrix” was the word that came to mind. As she said, she is the one getting the system to behave as she wants it to. Even if she allows it to make decisions, she is monitoring it and will not always follow its suggestions. Reflecting on, evaluating, and making changes to the loop are what is described as essential to making the loop work and valuable for the users. As Wiedemann (2021) argues, looping requires constant and mutual attentiveness between the person, the body, and the technological devices. Our analysis shows that this does not only involve the user as a maintenance worker in charge of repairing and fixing the physical and material aspects of the ADM system so devices can stay attentive to bodily and technological signals. It also involves the user working as a reflexive being who needs to stay attentive to what the ADM system is communicating. In our case, the user is in charge of monitoring, analyzing, reflecting on, and modifying data input as well as the system’s setting based on personal experiences, circumstances, and bodily states and emotions. Thus, the above examples all testify to how modification and anticipation in turn-tacking with the system are practices that are lived, sensed, and felt in both physical and emotional ways by the users who are embodying these systems throughout their daily lives.

## Concluding discussion

People who loop can attest to the common objection to hype about AI and automated systems that “*automation is not really automated*” (Forlano 2023b; see also Forlano 2023a and Crawford 2021). As we have seen in the analysis, automation rests on human engagement and effort at multiple instances from system development, data collection, and training to the actual everyday use with which we are concerned here. Still, to fully recognize this and account for the human involvement and efforts entailed in everyday ADM, we need to conceptualize how, more specifically, humans and ADM together produce what we have come to call automation. In this paper, we have argued that the concepts of turn-taking and anticipation can help us qualify how people enable the continuous circuit necessary for providing them with insulin to manage their diabetes. In looping, it is not so much the body which has to be cared for as the system. Our analysis thereby confirms what Wiedemann (2021) describes in current diabetes management as a move from caring for the body to caring for technology. At the same time, we extend this finding by showcasing not only how the human is positioned as an active maintenance worker, but also as a modifier of decision-making processes with the DIY APS system as an empirical example of everyday ADM.

Specifically in our material, we have found two narratives of caring for the loop. The first narrative described practices of caring for the loop as a stable communication circuit for the algorithm to make decisions based on bodily states and fluctuations. In this case, humans had to ensure proper connection between the body and the devices, as well as in between devices. Such practices had the purpose of facilitating the physical and material base for efficient turn-taking in the internal communication circuit connected with their body. This meant dealing with actual or anticipated failures or other forms of breakages that would cause gaps in the synchronous turn-taking, such as devices falling off the body, being held at a distance too far to maintain the connection, or losing connection due to external factors. While these care practices contributed to the normalization of disease management by helping users divert their focus from diabetes to the technical ecosystem itself, the maintenance practices and efforts being invested in keeping the system going were made invisible to the outside world, as they were seen to be incrementally part of mundane everyday routines. The second narrative uncovered in the analysis was related to the previous narrative and concerned users’ asynchronous involvement as reflexive beings in the turn-taking with the system. This narrative described practices of caring for the loop through the continuous work of overseeing and modifying it based on human reflections. These concerned data visible in the Loop app, as well as external factors that could be influencing the communicative loop, such as eating certain foods or increasing the level of physical activity. Modifications of the loop were seen, for instance, when users interfered with the system’s settings, added, or ignored certain data points, and made sure to always stay aware and inform the system of bodily states, feelings, and outside circumstances that they anticipated could affect the loop. Despite users caring for the system through both maintenance and modification practices, they still encountered system failures or mismatches in the suggestions it made, which sometimes prompted them to overrule the system’s calculations by ignoring certain suggestions or, on occasion, even turn off the system completely. Thus, on the one hand, looping introduces a range of new sources of possible errors circulating around the body in the case of diabetes management. These errors often require the user to first turn the focus towards fixing the technology. On the other hand, users view these errors as inseparable parts of what it means to engage closely with Loop and almost use it as an entrance into personalizing their own use of the system.

Taking looping as an example of everyday ADM, the analysis provides insight into what automation might look like today and how systems we take as being automated “[…] require constant human efforts to become what they end up being” (Ruckenstein 2023: 1242). In looping, what is understood as automation is a system that requires human attention, turn-taking, engagement, and effort to function down to the very detail of how it operates. Thus, what we might understand by involving the human in the loop in empirical reality concerns human involvement in maintenance and modification work hidden in small everyday practicalities of communicating with personal devices. Here, it is important to note that the system we have studied is an “open closed loop system”, meaning that users’ ways of being in and caring for the loop are conditioned by open source: Users have forced themselves access to the coding of the loop and thereby to their data. Had they instead been users of a commercial “hybrid closed loop system”, we would have potentially heard narratives characterized by less maintenance and modification work, but with the possible risk of other problems arising, such as irrelevant alarms, lack of interoperability between devices, and limited customization of personal settings. As Forlano (2023b; n.p.) argues, we can choose to “adopt a positive relationship to these anticipated technological breakdowns” and instead see maintenance of technological breakdowns “in more relational terms” with humans. This approach means viewing humans and technology as two entities that can both fail and succeed together “[…] if we reject the myths of objectivity, perfection and solutionism that tend to pervade our current debates around AI systems” (Forlano 2023b: n.p.). In line with this is the understanding that “technology *itself* is disabled—full of flaws and failures, gaps and glitches, seams and symptoms, errors and omissions, bugs and biases” (Ibid.). Such systems can be considered disabled in several ways, including when there are software or hardware issues, when they fail to integrate with broader societal contexts they are embedded within, and when people either refuse or reimagine them (Forlano 2023a). In line with this, as our analysis indicates—potentially at a more basic level—that succeeding with everyday ADM, in the case of looping, requires humans to first and foremost navigate the fine line between knowing when to engage with the technology and when not to. Namely, the communicative process of engaging relationally with the system by caring for it through maintenance and modification work places huge demands on humans for their labor just as much as humans demand certain things from the system.

## Data Availability

The data associated with this study is not publicly available due to confidentiality restrictions.

## Footnotes

1 With the latest technological developments there are now systems that do not depend on MiaoMiao and RileyLink.

2 The data generated and analyzed during the current study are not publicly available to ensure the anonymity of participants, but can be made available in anonymized form from the main author on reasonable request.
